# Bubonic Plague at the Cape

**Published:** 1901-04-13

**Authors:** 


### The Hospital

A Journal of

The Medical Sciences and Hospital Administration.

Vol. XXX.—No. 759.] Edited by Sir Henry Burdett, K.C.B., and Solomon C. Smith, M.D., M.R.C.P. [April 13, 1901.

### BUBONIC PLAGUE AT THE CAPE.

Few things could testify more strongly to the tranquilising effect of knowledge than the comparatively small amount of attention which has been excited by the appearance of bubonic plague in South Africa, and especially at Capetown. We read from day to day, interspersed among the telegrams which announce the successive evanescences of De Wet, or the captures of General French, or the murder in cold blood of native women and children by the Boers, others which inform us that there have been such or such a number of cases of plague, so many of them among Europeans, and that such or such a proportion has terminated fatally. We sometimes read also the almost superfluous declaration that proper precautions are being taken by the local sanitary authorities; and we pass on to the next paragraph with scarcely more concern than would be produced by the announcement of a score or so of cases of mumps or of measles. In truth we have learnt, not only what Sir John Simon taught many years ago, that, under modern conditions of travelling and of commerce, contagions current in the East must be expected to become current in Europe, but we have also learnt, thanks partly to the laboratory investigations of Pasteur and his fellow-workers and successors, partly to the application of the results of their labours by sanitary reformers, that contagions are more easily to be guarded against, and are less to be dreaded, than it entered into the minds of our forefathers to suppose. With better knowledge of the material causes and modes of propagation of infections, irrational terrors are everywhere giving place to reasonable precautions; and these again are adjusted to the particular forms of malady which they are intended to combat. We may still occasionally see a sprinkling of pink powder, as if it had been scattered from a flour dredger, adorning the upper stratum of some heap of decaying refuse, or the margin of a sewer outlet; but, in a general way, once more to quote Simon, the "vague chemical libations and powderings" formerly held to constitute what was called "disinfection" have disappeared into the limbo of the well-nigh forgotten past. Our precautions are directed towards the particular evil against which they are designed to provide, and, having once taken them, we are able to trust calmly in their general efficiency. Epidemic disease, will not be prevented until all mankind learn to exercise a prudence guided by knowledge; but, in the meantime, it is at least shorn of its terrors for the great bulk of the community.

With regard to any particular form of epidemic, moreover, it is of extreme interest to observe its unquestionable tendency to die out in particular communities, more especially after a period of extreme prevalence or of great severity. Plague occurred sporadically in England for many years previous to the great epidemic of 1665, which preceded its disappearance from the country; and in France for many years previous to the outbreak of 1720 at Marseilles, which also was final. We are now able to regard occurrences of this kind, which were unintelligible when they were first observed, as the results of acquired national immunity, although the sources of this immunity are still unknown to us, and are left to furnish matters of research for the bacteriologists of the future. We learn from various Indian sources that acquired immunity is becoming manifest among rats, great numbers of which, in all probability, have, so to speak, conducted experiments upon themselves, by the accidental ingestion of small doses of the poison, from the effects of which they have recovered. How far such immunity can be artificially produced, how long it will last, and in what degree it can be transmitted to offspring, are questions of primary importance which at present await solution; while the experience of India, during the next few years, may be expected to throw light upon the equally important question of the acquired immunity of a population among which a particular disease has affected a certain percentage of persons, and then seems to be destitute of power to affect the rest. It is said that Indian sanitarians, in view of the lingering of plague in certain localities, and of the difficulty or impossibility of enforcing the precautions which would be taken against it in any less complicated state of society, are on the whole disposed largely to put their trust in the hope of that natural period of extinction which history encourages us to expect in the fulness of time. The sanitary conditions of South Africa, in the meanwhile, are being subjected to the careful scrutiny of very capable observers. A Commission of competent investigators has been strenuously at work, one of its members being Dr. Simpson, formerly Medical Officer of Health for Calcutta. When the report of this Commission is before the public we shall at last have trustworthy information on the chief sanitary aspects of the war, and also, in all probability, upon the sanitary state and requirements of the colony. It will be of special interest to see how far the Commissioners can elucidate the problem of the methods of diffusion of enteric fever, a diffusion which many South African observers have attributed to dust and flies, rather than to the polluted water which is chiefly blamed when such diffusion occurs among European communities.

### A Hospital for the Unborn!

According as one does or does not believe in heredity as the main factor by which the progress or the decadence of the race is determined, so is one inclined to regard critically or kindly the efforts of physicians to cure the sick. For humanity's sake, in the cause of charity, and, it may be added, with a laudable desire to earn a living, we do our best to cure diseases and cocker up sick folk. Yet if we look beyond the individual and consider the race, we can hardly doubt that all this tinkering of invalids is a direct interference with well recognised "laws of Nature," and that just so far as we succeed in setting on their feet those who, without our aid, would have been eliminated as "unfit," we lower the racial stamina and spoil the breed. But the question must sometimes arise whether this duty of saving life, which we have undertaken as our daily task, may not be carried to an almost undue extreme, and probably many will think that this question very properly arises in regard to the proposal to found hospitals for the unborn! No one who recognises how largely the dangers of childbirth are due to conditions which might be discovered, and might indeed sometimes be guarded against, were the expecting mother to be under medical care for some time before her hour of trial, need hesitate in admitting that in hundreds of cases it would be the kindest of charities to take these women into well organised hospitals or homes, where, during the later months of their time, they might at the least have rest, and where steps might be taken to minimise the risk of what every woman regards as the most dangerous event of her life. But when we are urged to found such hospitals on the plea that they would tend to the conservation of fœtal life, that is of fœtal life which Nature would destroy, the matter assumes a somewhat different aspect. There are certain diseases and deformities and monstrosities which Nature eliminates by the simple method of preventing their propagation. For the sake of the race it is well that this should be so, and the question is, Ought we to interfere? In regard to sentient mankind we put on one side all considerations of race and posterity, and in the name of humanity do our best to relieve suffering. But in regard to the unborn, ought we to help these immature, deformed, and sickly ones to pass the portal at which Nature would destroy them, and let them come into the world carrying with them, as we believe, all their tendencies to produce immature and deformed and sickly offspring in their turn? When as an illustration of the sort of case in which such a hospital might be used we are told of a woman who was truly a "monstripara," a producer of monsters, a woman who had already brought three monstrous fœtuses into the world and had had several abortions, we may well ask whether it is wise, even were it possible, to interfere with Nature's ways of preventing the multiplication of "sports" and monsters. Let the poor woman stand aside and let the task of peopling the world be left to those who are more fitted for it.

### Sanitary Mediævalism.

York has long been unduly subject to enteric fever, and its liability in this respect seems to be due to some influence other than water supply. In an ancient city like York, the area within the castellated walls of which has been continuously built upon for a couple of thousand years and more, and in which for many hundreds of years the houses have, as in all walled cities, been crowded together with narrow streets and small backyards, it is not to be wondered at that the porous soil has become so contaminated as to form a suitable nidus for the development of the bacillus typhosus. With the introduction of better drainage and water supply, the influence of such a polluted soil ought gradually to disappear. But in York outbreaks of typhoid keep recurring, and Dr. Edmund M. Smith, the medical officer of health, throws the blame on the persistence of that old relic of mediævalism the midden-privy. Taking the whole of the cases into consideration he says that the majority were associated with the existence of midden-privies, most of which were more or less foul or leaking, with uncemented walls and floors; in not a few instances with dilapidated walls, most of them permitting of the pollution of the adjacent soil. A large number of them were inches deep in liquid filth or so full of refuse as to reach above the cemented portion of the walls. A very striking fact in regard to the association of enteric fever with this mediæval method of conservancy is to be found in Dr. Edmund Smith's report, namely, that out of a total of thirteen houses affected with typhoid fever during the year 1900 which had been before affected in the same way during the previous six years, twelve had midden-privies. If one wants to keep typhoid infection constantly on tap for the use of oneself and one's friends, evidently a midden-privy near the kitchen door is just the thing. The midden is the reservoir of the infection, while cats, mice and flies serve as the mechanism for its distribution.

### The Permissible Dose of Arsenic in Food.

In the proceedings before the Royal Commission which is investigating the subject of arsenical poisoning, a question has several times arisen as to how much arsenic may be consumed with impunity. It has become very clear that traces of arsenic are liable to be far more widely present in food products than has hitherto been suspected, and the questions How much is to be admitted? and What is to be the meaning of the phrase "a mere trace of arsenic?" so often used by chemists, become matters of considerable importance. We have on several occasions referred to the very considerable quantities of arsenic which are sometimes taken in the form of medicine without harm resulting, and in his evidence before the Commission, Professor Delépine reverted to the same point in regard to the drinking of mineral waters, showing that during a course of Vichy waters a patient might take a daily dose equivalent to quantities of arsenious acid ranging between 1·224 and 1·45 grains with benefit. He said that considering the large number of people who had taken these waters without any symptoms of arsenical poisoning being detected, it was probable that a quantity of one-sixtieth of a grain of arsenic per gallon of beer was harmless. But he added the important proviso "provided that the arsenic was not present in a form more poisonous than arsenious acid." This, however, is begging the question, for it is just in regard to the exact form in which arsenic exists in such an organic preparation as beer, and as to the comparative toxicity of the organic preparations of such metals as arsenic, that we know so little. On every side the pharmacists are asserting that organic preparations of the metals possess an activity far greater than the crude inorganic salts. Every art of the advertiser is being employed to induce us to order silver, mercury, and especially iron, in organic combinations, and although physicians of the older school still swear by the old formulæ, and order their tinct. ferri perchlor. as in days gone by, there are plenty who side with the modern pharmacists, and maintain that by organic combinations we can get effects which are unattainable by the use of inorganic salts. If true of such drugs, this ought to be true also of arsenic, and under these circumstances we do not like to accept too readily the suggestion that certain quantities of arsenic in articles of diet may be regarded as harmless, merely because certain equivalent quantities of the metal are known to be often taken without harm.

### Wooden Houses.

It is a misfortune that building bye-laws are mostly framed with reference to the requirements of urban districts, for much useless inconvenience is often entailed upon those who live in the open country by the fact that they have to submit to regulations devised to meet requirements very different from their own. As a marked example of the disabilities so entailed upon country dwellers in some districts, we may instance the prohibition of wooden houses for purposes of human habitation—attention to which has lately been drawn by a deputation of architects having waited on the central authorities in reference to the subject. It seems reasonable to believe that a stone house, or one constructed of brick with hollow walls, will give more complete protection from the vicissitudes of the weather than a house merely built of wood, but we are not certain even of this. There however can be no question that a wooden house properly raised above the level of the ground, as all wooden houses are likely to be for their own preservation, may be made a far drier and more healthy residence than the majority of country cottages, built as they are at present, can ever become. We are beginning to understand, what our forefathers knew perfectly well, although perhaps they did not fully understand, that in house construction dryness and warmth are the main points, and as to both dryness and warmth a frame house properly boarded or plastered without and within, so as to leave air spaces between the inner and the outer layer, and so arranged that the air shall circulate freely under the floor, is far superior as a residence to an ordinary brick building, especially when this is arranged, as such buildings so often are, without any air spaces in the thickness of the walls or beneath the flooring. The only valid objections to such houses are fire and vermin. But as to spiders the same applies to all cottage roofs; and as to fire, wooden houses have this great virtue that they are never high. A sitting-room on the ground floor and a chamber above is as high as any such houses are likely to go, and we venture to say, speaking, of course, only of isolated cottages in the country, that their inmates are in less danger from fire than those who dwell in tall brick houses, from which there is no escape except at the risk of a broken neck. No one would think of building a row of wooden houses, or of using wood in towns where one fire might become the centre of a conflagration; but it does seem a pity, when the difficulty of finding healthy cottages is so great, that any obstacle should be placed in the way of inventors who, if the field were open, would doubtless be able to discover some cheaper way of building houses than by piling up ton after ton of absorbent bricks. Thickness of wall is nothing but a disadvantage when it is more or less saturated with moisture, which must be evaporated before the warmth of the sun can penetrate.

### The British Congress on Tuberculosis.

The British Congress on Tuberculosis, which will be held in the Queen's Hall, London, from Monday, July 22nd to Friday, July 26th, under the patronage of His Majesty the King, will be an event of considerable importance. Among the various matters to be discussed by the State and Municipal Section are questions touching the geographical distribution of tuberculosis; its incidence in particular occupations; its distribution in the different parts of London; its relation to soil, to milk and meat supply; questions regarding the influence of housing and of aggregation; and regarding the notification of tuberculosis and the provision of sanatoria for phthisical patients. The Medical Section will deal with questions of climate, of sanatoria, and with the uses of tuberculin in diagnosis and in treatment. A special section will be devoted to Pathology, including Bacteriology, in which it is expected that eminent professors from many countries will discuss the origin and processes of the disease; and finally there will be a Veterinary Section, dealing with the many thorny problems which arise in relation to tuberculosis in animals. When one reads over the list of subjects to be discussed one cannot but feel impressed with the extent and importance of the topics which are to be brought forward, and of the interests which are involved, and we are glad to note the very large infusion of the medical element in all the sections. The recent popularisation of the whole question, while it has done much good in lifting the subject of the prevention of tuberculosis out of the academic rut, has, on the other hand, now and again led self-confident laymen to rush in even where doctors feared to tread, and there undoubtedly has been a danger of the Congress ending in a mere repetition of popular views at second hand. There is every reason to believe, however, that this danger has been avoided, and that some real interchange of opinion between real investigators will take place. As, however, with all congresses, the great points will be the social intercourse between leaders of thought; the wider popularisation of the idea; and the *cachet* given to it before the public by great names.

### Indian Asylums.

The new scheme for the creation of five large central asylums for the insane in India in place of the numerous small asylums which have hitherto been under the care of civil surgeons, with no claim to special knowledge of psychology, is a step in the right direction, and the appointments to be made in connection with them will no doubt prove an attraction to the Indian Medical Service, from which the medical officers to the asylums will probably be selected. One great danger in all such institutions lies in their tendency to grow to undue dimensions, and it is to be feared that this is a risk which will be especially great in India with its immense population; but if this can be avoided, and if they can be prevented becoming mere refuges for destitute imbeciles, and for the mental and religious "freaks" which abound in that country, the new asylums ought to be a great improvement on the old ones.

